# Infections néonatales: quelle est la place des antécédents obstétricaux dans la prévention du risque?

**DOI:** 10.11604/pamj.2014.19.133.4432

**Published:** 2014-10-07

**Authors:** Adonis Nyenga, Olivier Mukuku, Augustin Mulangu Mutombo, Oscar Numbi Luboya

**Affiliations:** 1 Faculté de Médecine, Université de Lubumbashi, République Démocratique du Congo; 2 Ecole de Santé Publique, Université de Lubumbashi, République Démocratique du Congo

**Keywords:** Infection, néonatale, mortalité, antécédents obstétricaux, Lubumbashi, Infection, neonatal, mortality, obstetrical history, Lubumbashi

## Aux editeurs du Journal Panafricain de Médecine

La majorité de ces décès néonatals est due aux infections (30-40% contre 27% pour la prématurité et 23% pour les détresses respiratoires) parmi lesquelles la gravité des infections néonatales bactériennes est connue. L’étude prospective que nous avons menée dans l'unité de néonatologie des Cliniques Universitaires de Lubumbashi sur une période allant du 1^er^ août 2011 au 31 juillet 2012 dans le but d'attribuer une valeur prédictive aux différents facteurs obstétricaux de risque infectieux dans la survenue de l'infection chez le nouveau-né nous montre qu'il existe une association significative entre l'infection néonatale et les antécédents obstétricaux suivants: l'infection urogénitale non traitée au cours du dernier mois de grossesse (OR = 26,40; IC95%: 3,34-208,63), la rupture prématurée des membranes de plus de 12 heures avant l'accouchement (OR = 14,71; IC95%: 4,77-45,34) et l'absence de surveillance prénatale de la grossesse (OR = 2,55; IC95%: 1,35-4,83). C'est ainsi qu'une identification des nouveau-nés à risque sur base de ces antécédents obstétricaux maternels permettrait une intervention en amont et donc une approche efficiente dans la prévention des conséquences liées à l'infection du nouveau-né.

Selon l'OMS, l'infection néonatale est responsable de près de 30 à 40% de la mortalité néonatale dans les pays du tiers monde [1]. Plusieurs facteurs contribuent à maintenir élevée la mortalité liée aux infections néonatales (INN) au rang desquels on retrouve, non seulement le manque de suivi des consultations prénatales (CPN), mais aussi l'accès réduit aux explorations paracliniques pouvant préciser le diagnostic étiologique [2]. C'est ainsi qu'une identification des nouveau-nés à risque sur base des antécédents obstétricaux des mères permettrait une intervention en amont et donc une approche efficiente dans la prévention des conséquences liées à l'infection du nouveau-né. La présente étude, avait pour objectif d'attribuer une valeur prédictive aux différents facteurs obstétricaux de risque infectieux.

Cette étude est prospective analytique menée dans l'unité de néonatologie des Cliniques Universitaires de Lubumbashi sur une période allant du 1^er^ août 2011 au 31 juillet 2012. L’étude a porté sur 62 nouveau-nés admis pour INN (groupe I). Des paramètres obstétricaux de risque infectieux materno-transmis ont été analysés en comparaison avec 111 nouveau-nés admis dans l'unité de néonatologie pour un diagnostic autre que l'INN (groupe II) au cours de la même période. Les analyses statistiques ont été réalisées sur le logiciel Epi Info 2011 (version 7.0.8.3) et les Odds ratio calculés avec un intervalle de confiance à 95%. Le seuil de signification a été fixé à p < 0,05.

Comme nous le montre le Tableau 1, nous avons noté une association statistiquement significative entre certains antécédents obstétricaux et la survenue de l'INN (p

**Tableau 1:** Risque infectieux en rapport avec les antécédents obstétricaux

Paramètre	Groupe I (n = 62)	Groupe II (n = 111)	OR	IC_95%_	p
Absence de surveillance prénatale de la grossesse	36 (58,1)	39 (37,1)	2,55	1,35-4,83	0,0058
Infections urogénitales maternelles	12 (19,4)	1 (0,9)	26,40	3,34-208,63	0,0000
Age gestationnel ≤ 37 SA	37 (59,7)	86 (77,5)	0,43	0,21-0,84	0,0213
Rupture prématurée des membranes >12 heures	22 (35,5)	4 (3,6)	14,71	4,77-45,34	0,0000

SA: semaines d'aménorrhée; OR: odds ratio; IC_95%_: intervalle de confiance à 95%

La RPM se trouve être le facteur de risque le plus retrouvé dans la littérature avec une association statistiquement significative entre celle-ci et la survenue de l'infection. En effet, la rupture de la poche des eaux entraîne un risque élevé des infections foetales dont la fréquence est multipliée par 10 à 100 après 24 heures [3]. Il est en outre établi une relation entre l'infection uro-génitale et le risque de rupture avant terme des membranes [4]. L'insuffisance de la surveillance de la grossesse est associée à une morbi-mortalité néonatale et maternelle élevée [5]. Ainsi, il découle de ces constats que l'INN ne devrait pas être considéré comme une fatalité étant donné que des actions efficaces et moins couteuses en avale pourraient permettre la prévention de celle-ci. Il s'agit essentiellement d'une surveillance qualifiée de la grossesse par des consultations prénatales bien conduites et une hygiène de vie conséquente au cours de la gestation. Ceci laisse entrevoir qu'en améliorant l'accessibilité aux soins prénatals et en misant sur une éducation sanitaire des femmes sur le comportement à moindre risque pendant la gestation, on pourrait améliorer la morbi-mortalité néonatale par la réduction de la fréquence des INN bactériennes.

En conclusion, il est donc utile que toute femme enceinte soit suivi de façon optimale afin de prévenir et de prendre en charge correctement les facteurs de risque infectieux et que toute femme en âge de procréer soit informer sur les comportements à moindre risque au cours de la gestation.
